# Clinical and procedural outcomes of very high-power short-duration vs. standard power ablation for pulmonary vein isolation

**DOI:** 10.3389/fcvm.2026.1806509

**Published:** 2026-04-21

**Authors:** Inês Rodrigues, João Almeida, Rafael Teixeira, Tiago Martins, Mafalda Carrington, Helena Gonçalves, João Primo, Marco Oliveira, Paulo Fonseca, Ricardo Fontes-Carvalho

**Affiliations:** 1 Cardiology Department, Unidade Local de Saúde Vila Nova de Gaia/Espinho, Vila Nova de Gaia, Portugal; 2 CINTESIS@RISE, Department of Community Medicine, Information and Health Decision Sciences, Faculty of Medicine, University of Porto, Porto, Portugal; 3 Cardiology Department, Hospital da Luz Arrábida, Vila Nova de Gaia, Portugal

**Keywords:** atrial fibrillation, catheter ablation, high-power short-duration, pulmonary vein isolation, radiofrequency

## Abstract

**Introduction:**

Pulmonary vein isolation (PVI) using shorter but higher-power radiofrequency (RF) applications can improve lesion quality and reduce procedural time compared to the standard approach, while ensuring similar clinical and safety outcomes. The aim of this study was to compare very high-power short-duration (vHPSD) with standard-power atrial fibrillation (AF) ablation regarding clinical outcomes, procedural efficiency and safety.

**Methods:**

All patients who underwent first PVI using RF energy between January 2021 and June 2024 were included. The two groups (standard-power vs. vHPSD AF ablation) were matched using a propensity score analysis and compared at 12 months regarding freedom from AF recurrence, quality of life improvement and safety.

**Results:**

333 patients were submitted to PVI, and after the propensity score analysis, 226 patients were analysed (65.0% male, median age 62.8 years, comparable baseline characteristics). The median follow-up time was 554 days (IQR 351, 729) for the vHPSD group and 728 days (IQR 555, 730) for the standard group (*p* < 0.01). Freedom from AF recurrence at 12 months was similar between groups (83.7% vs. 86.1%, *p* = 0.85) and both groups exhibited significant and similar improvements in QoL measurements, following the procedure (mean adjusted difference of +13.9, 95% CI 10.4-17.5, *p* < 0.001). Total procedural time was significantly shorter in the vHPSD arm (86.5 min vs. 100.0 min, *p* < 0.01) and PV first-pass isolation (FPI) was significantly lower in the vHPSD group (52.3% vs. 72.3%, *p* < 0.01). AF catheter ablation had a favourable safety profile, with a low prevalence of adverse effects, irrespective of the type of RF energy used.

**Conclusion:**

VHPSD AF ablation was a more efficient technique with a shorter procedural time, achieving similar clinical outcomes despite having a lower FPI rate. Both techniques appeared to be safe with low prevalence of adverse effects.

## 1 Introduction

Atrial fibrillation (AF) is the most common cardiac arrythmia. Over the past few decades, AF catheter ablation has become an evidence-based, safe, and effective treatment. The procedure primarily focuses on pulmonary veins’ electrical isolation from the left atrium (LA), frequently using radiofrequency (RF) energy (1–3).

Despite its effectiveness in reducing symptoms and slowing AF progression, 12-month AF recurrence after an ablation procedure can still reach 15 to 30% in the most recent studies (4, 5), mostly due to suboptimal lesion formation and consequently pulmonary veins (PV) reconnection (6).

New techniques and catheters using shorter but higher-power RF applications have the potential to improve lesion quality and durability (2). These methods are expected to increase the resistive form of tissue heating, which causes immediate and irreversible cell injury, while minimizing conductive heating that passively extends to adjacent tissues potentially leading to unwanted damage (6). As a result, wider and shallower lesions are produced, ensuring better lesion contiguity while maintaining sufficient depth to achieve transmurallity (2, 6). Applying RF energy for shorter duration time also minimizes catheter instability, which is another factor contributing to suboptimal lesion formation (7).

Previous evidence has suggested that very high-power short-duration (vHPSD) AF ablation can reduce procedural time with similar results regarding safety and AF recurrences, compared to the standard approach (7–11). However, published studies to date often include limited sample sizes and follow-up periods restricted to one year.

The aim of this study is to compare vHPSD AF ablation with a matched control cohort undergoing standard power AF ablation in a high-volume centre regarding clinical efficacy outcomes, procedural efficiency and safety.

## 2 Material and methods

### 2.1 Study population and design

This was an observational, retrospective study that included all patients aged 18 years or older with paroxysmal or persistent AF, who underwent first pulmonary vein isolation (PVI) at our center between January 2021 and June 2024. Procedures using non-RF energy were excluded.

Standard power AF ablation was performed until April 2022, after which vHPSD AF ablation became the standard approach through the end of the study. The two groups were subsequently matched using a propensity score analysis and compared with respect to clinical outcomes, procedural efficiency, and adverse events (Supplementary Figure 1).

### 2.2 Procedure description

All patients were instructed to take oral anticoagulants for one month prior to the procedure and to continue this treatment for at least three additional months thereafter. Cardiac computed tomography (CT) was performed before the procedure to rule out intracardiac thrombus and to identify any anatomical abnormalities. All procedures were conducted under general anaesthesia, and the CARTO 3 system was used for electroanatomic mapping in every case. Unfractionated heparin was administered in order to maintain an activated clot time above 300s. Standard-power AF ablation was performed using 35-watt RF applications guided by Ablation Index (AI) (>500 for anterior segment and > 380 for the remaining segments), using the Thermocool SmartTouch SF Catheter, with a fixed curved sheath (SL0). In selected cases (e.g., markedly dilated LA or catheter instability), a deflectable sheath could be used at the operator's discretion. vHPSD catheter ablation was performed using the QDOT Micro ablation catheter, with a deflectable sheath – Agillis or Vizigo. In this group, 90-Watt, 4-second RF applications were applied in all regions, except the anterior region, where 50-watt RF applications guided by AI (>500) were used. In both strategies, interlesion distance was <6 mm. Procedural success was defined as the complete electrical isolation of all PV. Additional lesions were executed at the operator's discretion.

### 2.3 Follow-up program

Patients were included in a two-year digital follow-up program, conducted through a dedicated web platform: Promptly - Software Solutions for Health Measures (https://promptlyhealth.com). This platform allowed physicians to enter baseline demographic data, as well as to record procedural and follow-up outcomes. It also provided access to patient self-reports in real time. For patients, the platform allowed them to self-report symptoms, adverse events, and vital signs at any time. Additionally, patients could upload electrocardiograms performed externally, in other ambulatory clinics. The platform was also used to complete standardized healthcare questionnaires.

In addition to the digital follow-up, all patients were evaluated in telephone consultations at 1, 3, 6, and 18 months and were required to attend in-person consultations at 12 and 24 months. All consultations were conducted by cardiac electrophysiologists, fellows, or cardiology residents.

Patients were instructed to obtain a standard 12-lead electrocardiogram (ECG) at 3 and 6 months and upload the results to the digital platform for physician review. A 24-hour Holter electrocardiogram was required between months 9 and 12 of follow-up. Additionally, ECGs were systematically performed during scheduled in-person visits at 12 and 24 months to assess cardiac rhythm.

### 2.4 Endpoints

Patients were followed for two years after the procedure. The primary efficacy outcome was freedom from AF recurrence at 12 months. Recurrences were defined as any ECG-documented atrial tachyarrhythmia lasting at least 30 s, including AF, atrial tachycardia, and atrial flutter. The first two months following PVI were defined as the blanking period, and any arrhythmias occurring during this period were excluded from the primary endpoint analysis.

Two additional composite endpoints were evaluated: 1) new antiarrhythmic intervention, defined as the occurrence of any of the following – redo ablation procedure, electrical cardioversion or antiarrhythmic drugs (AADs) reintroduction; and 2) healthcare utilization, which included emergency department (ED) visits due to AF or congestive heart failure (CHF); hospitalizations related to CHF, AF, acute coronary syndrome (ACS) or stroke; electrical cardioversions; and redo ablation procedures.

The secondary endpoint was quality of life improvement, assessed using the Atrial Fibrillation Effect on QualiTy-of-Life (AFEQT) score (12), a validated tool for evaluating AF-related symptoms and quality of life. A change of 5 or more points in this score was considered clinically meaningful.

Procedural efficiency outcomes included procedure and RF application times, and first-pass PVI rate. First-pass isolation (FPI) was defined as the acute inhibition of conduction between the LA and ipsilateral PVs during the first round of circumferential ablation.

Safety was additionally assessed by recording procedure-related complications including vascular events, pericardial effusion or tamponade, pericarditis, and stroke or TIA.

### 2.5 Statistical analysis

Baseline characteristics and clinical and procedural outcomes are expressed as absolute and relative frequencies for categorical variables; and as mean and standard deviation or as median and interquartile range for continuous variables, as appropriate. Categorical variables were compared using the chi-square test and Fisher's exact test. The Mann–Whitney test or two-sample t-test were used to compare continuous variables. Assessment of normality was performed by graphical visual analysis (histograms and QQ-plot).

Propensity-score matching (1:1 match using the nearest-neighbour method) was performed to reduce confounding bias. To account for potential temporal confounding related to the different study periods, calendar time was incorporated into the propensity score model as a part of a sensitivity analysis (further details provided in the Supplementary Material).

To compare primary outcomes between groups, a survival analysis was performed using Kaplan-Meyer curves and log-rank tests alongside Cox proportional hazards models with matching weights to adjust for the propensity score matching. Treatment effects were estimated with marginal hazard ratios and clustered-robust standard errors, accounting for dependencies within matched pairs. The AFEQT score was analysed using a linear mixed-effects model with scores as outcome variables and time, radiofrequency power, and interaction between time and radiofrequency power as fixed effects. Random effects included intercepts, adjusting for individual baseline differences. Restricted maximum likelihood estimation with an unstructured covariance matrix and Kenward-Roger degrees of freedom approximation was used. Mean differences with 95% confidence intervals (CI) between baseline and 12 months were calculated.

Statistical tests used two-sided *p*-values, and the significance level was 0.05.

Statistical analyses were performed using R statistical software 4.4.1 (Foundation for Statistical Computing, Vienna, Austria) and IBP SPSS Statistics 29.

## 3 Results

### 3.1 Baseline and clinical characteristics

A total of 333 patients underwent first PVI using RF energy between January 2021 and June 2024. After the propensity score matching, 226 patients were analysed, with 113 in each group. Most patients were male (65.0%), the median age was 62.8 years (IQR 54.2, 68.8), and the median CHA_2_DS_2_VASc was 1.5 (IQR 0, 3). They were more frequently referred for PVI due to paroxysmal AF (75.7%) and the median time since the initial diagnosis was 2 years (IQR 1.0, 4.0). Baseline characteristics before and after propensity score matching are summarized in Table 1.

**Table 1:** Description of baseline clinical characteristics before and after propensity score matching.

Variable	Before matching (*n* = 333)			After matching (*n* = 226)		
	vHPSD (*n* = 199)	Standard (*n* = 134)	*p*-value	vHPSD (*n* = 113)	Standard (*n* = 113)	*p*-value
Female	64 (32.2)	48 (35.8)	0.488	39 (34.5)	40 (35.4)	0.889
Age, years	61.5 (53.0, 67.6)	63.1 (55.9, 68.0)	0.362	62.7 (53.2, 68.3)	62.8 (55.7, 68.9)	0.799
Hypertension	81 (41.8)	77 (58.3)	0.003	56 (49.6)	60 (53.1)	0.595
Obesity	42 (22.2)	27 (20.9)	0.784	31 (27.4)	26 (23.0)	0.444
BMI, kg/m^2^	26.7 (24.4, 29.4)	27.5 (24.8, 29.3)	0.158	26.9 (24.7, 30.8)	27.4 (24.6, 29.7)	0.819
Smoking history	42 (21.8)	33 (25.0)	0.496	28 (24.8)	27 (23.9)	0.877
Type 2 diabetes	17 (8.8)	15 (11.4)	0.448	11 (9.7)	11 (9.7)	0.999
Sleep apnea	10 (5.2)	10 (7.6)	0.371	8 (7.1)	9 (8.0)	0.801
Thyroid disease	24 (12.5)	12 (9.1)	0.337	16 (14.2)	14 (12.4)	0.695
CAD	8 (4.1)	7 (5.3)	0.619	7 (6.2)	6 (5.3)	0.775
Heart failure	10 (10.5)	11 (8.5)	0.554	14 (12.4)	10 (8.9)	0.388
LVEF, %	59 (55, 61)	59 (56, 63)	0.027	59 (55, 62)	59 (56, 63)	0.297
Hemoglobin, g/dL	14.7 (13.5, 15.5)	14.6 (13.4, 15.5)	0.977	14.8 (13.5, 15.6)	14.6 (13.6, 15.6)	0.729
Creatinine, mg/dL	0.9 (0.8, 1.1)	0.8 (0.8, 1.1)	0.108	0.8 (0.8, 1.0)	0.9 (0.8, 1.0)	0.632
AAD			0.006			0.795
Class III	47 (25.0)	45 (34.1)		36 (31.9)	35 (31.0)	
Class Ic	26 (13.8)	30 (22.7)		20 (17.7)	24 (21.2)	
No	115 (61.2)	57 (47.2)		57 (50.4)	54 (47.8)	
HR medication						
Beta-blockers	113 (58.6)	97 (72.9)	0.008	81 (71.7)	78 (69.0)	0.662
Digoxin	4 (2.1)	2 (1.5)	0.999	4 (3.5)	2 (1.8)	0.683
AC type			0.720			0.999
NOAC	185 (95.9)	126 (94.0)		108 (95.6)	107 (94.7)	
Warfarin	3 (1.6)	3 (2.2)		1 (0.9)	1 (0.9)	
No	5 (2.6)	5 (3.7)		4 (3.5)	5 (4.4)	
LA dilation	124 (68.5)	96 (76.8)	0.113	82 (72.6)	86 (76.1)	0.542
LA vol, mL/m^2^	36 (29, 43)	37 (31, 47)	0.127	37 (30, 46)	37 (32, 47)	0.752
AF type			0.086			0.878
Paroxysmal	136 (69.0)	104 (77.6)		85 (75.2)	86 (76.1)	
Persistent	61 (31.0)	20 (22.4)		28 (24.8)	27 (23.9)	
Years since diagnosis	2.0 (1.0, 4.0)	2.0 (1.0, 4.0)	0.339	1.0 (1.0, 3.0)	2.0 (1.0, 4.0)	0.191

Values are shown as *n* (%) or median (IQR). AAD, antiarrhythmic drugs; AC, anticoagulation; AF, atrial fibrillation; BMI, body mass index; CAD, coronary artery disease; HR, heart rate; LA, left atrium; LVEF, left ventricular ejection fraction; NOAC, New oral anticoagulants; vHPSD, very high-power short-duration; vol: volume.

### 3.2 Procedural characteristics

Procedural data is summarized in Table 2. Ablation of all PV was successfully achieved in 96.5% of patients. Both total procedure time and RF application time were significantly shorter in the vHPSD group compared to the standard group (86.5 min vs. 100.0 min, *p* < 0.001; and 7 min vs. 24.5 min, *p* < 0.001, respectively). FPI was obtained in 52.3% of patients in the vHPSD group and 72.3% in the standard group (*p* = 0.001). Additionally, the standard group required a significantly larger volume of saline for catheter irrigation compared to the vHPSD group (529 vs. 200 mL, *p* < 0.001).

**Table 2:** Procedural characteristics.

Variable	vHPSD (*n* = 113)	Standard (*n* = 113)	*p*-value
Ablation type			0.410
PVI	84 (74.3)	80 (70.8)	
PVI+CTIa	24 (21.2)	23 (20.4)	
PVI+other	5 (4.4)	10 (8.9)	
Ablation of all pulmonary veins	110 (97.4)	108 (95.6)	0.722
Baseline rhythm			0.426
Atrial fibrillation/flutter	28 (24.8)	23 (20.4)	
Sinus rhythm	85 (75.2)	90 (79.7)	
Intraprocedural ECV	26 (23.0)	24 (21.2)	0.749
Total procedural time, min	86.5 (75.8, 100.0)	100.0 (86.0, 113.0)	<0.001
Ablation duration, min	32.0 (27.0, 38.0)	45.0 (38.0, 54.0)	<0.001
Total radiofrequency time, min	7.0 (5.0, 10.0)	24.5 (21.5, 29.9)	<0.001
Fluoroscopy time, min	5.0 (4.0, 6.0)	4.9 (3.5, 7.0)	0.847
Irrigation, mL	200.0 (174.3, 250.0)	529.0 (458.0, 638.5)	<0.001
First-pass isolation rate	67 (52.3)	94 (72.3)	0.001

Values are shown as *n* (%) or median (IQR). CTIa: cavotricuspid isthmus ablation; min: minutes; ECV: electrical cardioversion; PVI: pulmonary vein isolation; vHPSD: very high-power short-duration.

### 3.3 Clinical efficacy outcomes

**Figure 1:**
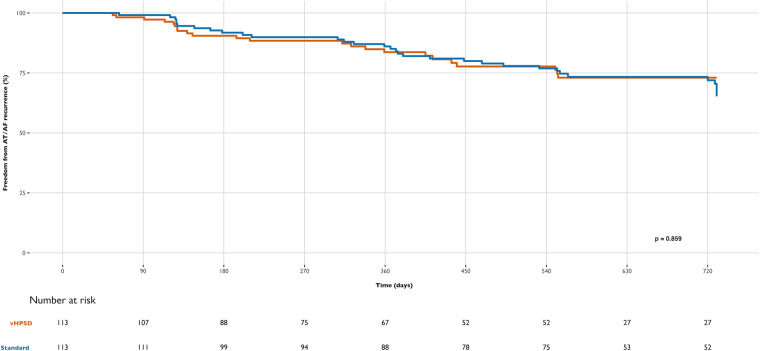
Differences in freedom from AF recurrence during follow-up between groups: very high-power short-duration (vHPSD) and standard power AF ablation.

**Figure 2:**
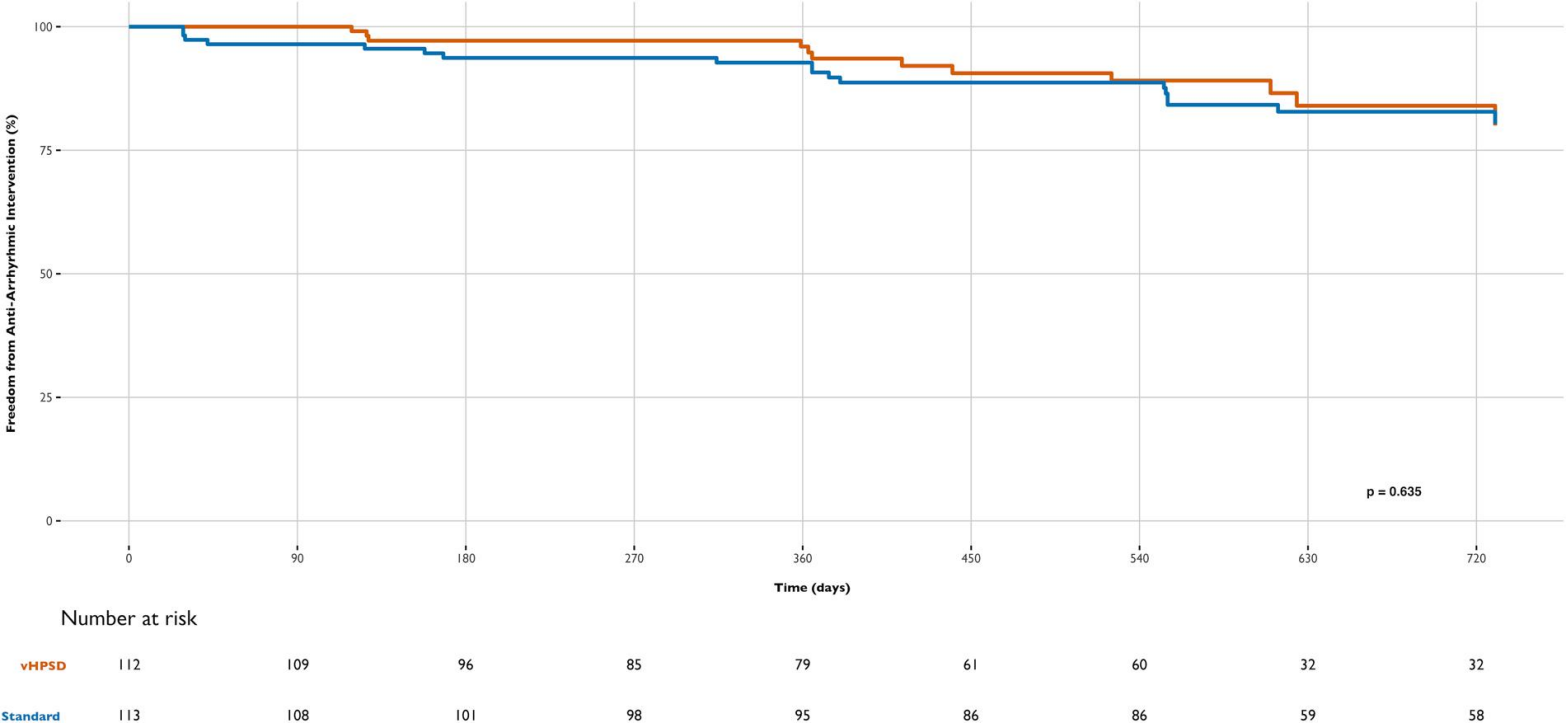
Differences in freedom from new antiarrhythmic intervention during follow-up between groups: very high-power short-duration (vHPSD) and standard power AF ablation.

**Figure 3:**
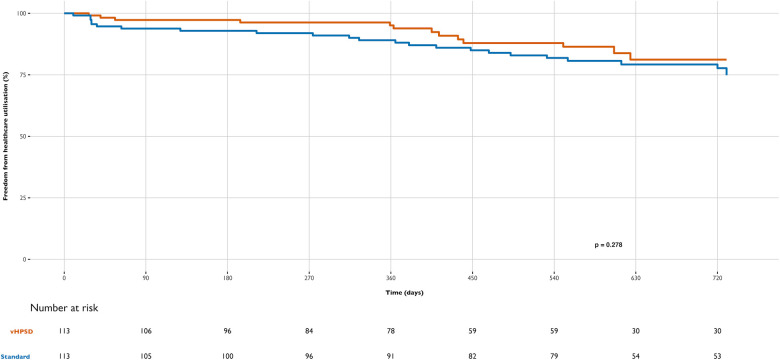
Differences in freedom from healthcare utilization during follow-up between groups: very high-power short-duration (vHPSD) and standard power AF ablation.

Patients were followed for two years after the procedure. The median follow-up time was 554 days (IQR 351, 729) for the vHPSD group and 728 days (IQR 555, 730) for the standard group (*p* < 0.0001). Clinical outcomes at 12 months regarding RF energy type are detailed in Table 3 and Figures 1–3.

**Table 3:** Clinical efficacy outcomes at 12 months.

Variable	vHPSD (*n* = 113)	Standard (*n* = 113)	*p*-value
Early recurrences (blanking period)	9 (8.0)	10 (8.9)	0.811
Persistent atrial fibrillation	6 (7.6)	12 (11.9)	0.342
Electrical cardioversion	5 (4.6)	7 (6.5)	0.353
Antiarrhythmic drugs reintroduction	5 (4.3)	4 (3.9)	0.883
Redo procedure	1 (1.0)	1 (0.9)	0.983
Emergency department visits	4 (3.7)	11 (9.3)	0.095
Hospitalizations^a^	0	0	NA
MACE	0	0	NA
All-cause mortality	0	0	NA

All results are presented as *n* (%). MACE, major adverse cardiovascular events (all-cause mortality, non-fatal MI, non-fatal stroke); vHPSD, very high-power short-duration.

a Hospitalizations related to congestive heart failure, atrial fibrillation, acute coronary syndrome, or stroke.

At 12 months, Kaplan–Meier curves and time-to-event analyses showed no significant difference in freedom from AF recurrence between groups (83,7% in the vHPSD group and 86,1% in the standard group; marginal HR 1.05, cluster-robust standard error of 0.26, *p* = 0.848) (Figure 1). During follow-up, most recurrences were symptomatic and primarily presented as paroxysmal AF in both groups. Additionally, no significant difference in AF recurrence was observed during the blanking period (*p* = 0.811).

New anti-arrhythmic intervention occurred in 6.5% of the vHPSD group and 9.3% of the standard group at one year, with no significant differences in Kaplan–Meier curves or time-to-event hazard ratios (marginal HR 1.19, cluster-robust standard error of 0.39, *p* = 0.647) (Figure 2). Similarly, there were no statistical differences between groups in the individual components of this composite endpoint.

Healthcare utilization at one year was also comparable between groups (6.1% in the vHPSD group and 11.9% in the standard group; marginal HR 1.46, cluster-robust standard error of 0.35, *p* = 0.277) (Figure 3). Although emergency department visits were numerically higher in the standard group, this difference did not reach statistical significance (3.7% in the vHPSD group and 9.3% in the standard group, *p* = 0.095). During follow-up, no hospitalizations due to CHF, AF, ACS, or stroke were recorded.

The sensitivity analysis adjusting the propensity score model for calendar time yielded results consistent with the primary analysis, with no significant differences in clinical efficacy outcomes between groups (Supplementary Material).

### 3.4 Quality of life

**Figure 4:**
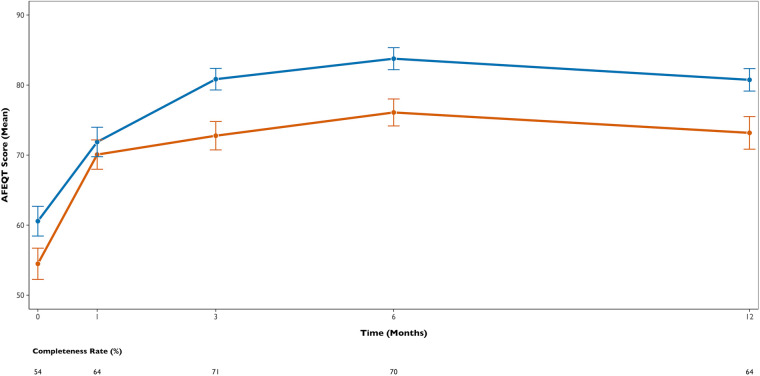
Comparison of quality of life measured by the Atrial Fibrillation Effect on QualiTy of life score (AFEQT) score. Completeness rates for each time point (questionnaires completed/sent) are displayed below the plots.

The median questionnaire completion rate per patient was 80% (completed/sent), with completion rates at specific time points ranging from 54% to 71% (Figure 4). Analysis with linear mixed-effects models showed a significant improvement in AFEQT scores over time in the entire cohort (mean adjusted difference between baseline and 12 months +13.9, 95% CI 10.4 to 17.5, *p* < 0.001) (Supplementary Table 1). Although patients submitted to standard power AF ablation tended to report slightly higher AFEQT scores overall (*p* = 0.058), no significant interaction between time and RF type was observed (*p* = 0.38), suggesting that the trajectory of quality-of-life improvement over 12 months was similar between groups.

### 3.5 Safety

Overall, AF catheter ablation has a favourable safety profile, with a low prevalence of adverse events, irrespective of the type of RF energy used (one pericardial effusion in the vHPSD group; one pericarditis and one vascular complication in the standard group) (Supplementary Table 2).

## 4 Discussion

In this study, vHPSD AF ablation was similar to the standard approach regarding clinical efficacy outcomes, including freedom from AF recurrence, need for new antiarrhythmic interventions, and healthcare utilization at 12 months. Additionally, Kaplan–Meier curves analysis showed no significant differences between groups in these outcomes over the 24-month follow-up. Both procedures effectively reduced symptoms and improved patients’ QoL. VHPSD AF ablation was associated with greater procedural efficiency, achieving similar PVI rates and clinical outcomes in a shorter procedural time, despite a lower FPI rate. Both procedures were safe, with a low prevalence of adverse events.

These findings align with previous reports (7–11, 13) and support them in a larger cohort from a high-volume centre during a two-year follow-up program.

PVI is the cornerstone of AF ablation (2) and was successfully achieved in 96.5% of patients in our cohort. In 3 patients in the vHPSD group and 5 patients in the standard group (*p* = 0.722) complete PVI could not be achieved. Incomplete isolation was mainly related to anatomical and procedural challenges, including unfavorable PV anatomy (mainly common left PV trunk or right intermediate branching variants) and persistent PV potentials despite multiple local RF applications, possibly related to tissue oedema, epicardial connections, and variations in LA wall thickness. These situations are well recognized in clinical practice and have been previously described (2). The vHPSD group exhibited significantly lower FPI rates, consistent with prior reports (7, 9). Differences in lesion geometry between ablation strategies may partly explain this observation. vHPSD RF applications are expected to increase resistive tissue heating while minimizing conductive heating, resulting in wider but shallower lesions, compared with standard power AF ablation (6). Recent evidence has suggested that, when compared with 50W applications, 90W applications may be less effective at achieving consistent transmural lesions, which could translate into lower FPI rates (14). However, lesion formation during RF ablation is often multifactorial and depends on several procedural and anatomical factors, including catheter stability, contact force and atrial tissue characteristics. Nevertheless, while previous evidence has suggested that FPI rates may predict AF recurrence (15, 16), our findings showed no significant differences in atrial arrhythmias recurrence rates at 12 and 24 months of follow-up, despite the lower FPI rate in the vHPSD group, suggesting that the final procedural global success may play a more important role in long-term outcomes than FPI alone. As reported in other studies (6–11, 13), the use of vHPSD RF applications significantly reduced both total procedural and RF application times, improving procedural efficiency. Additionally, shorter procedure duration may reduce LA dwelling times, potentially lowering thromboembolic events. Catheter saline irrigation is essential for maintaining adequate local temperature control during RF delivery (2). The lower irrigation volumes required with vHPSD catheters may eventually offer a hemodynamic advantage in patients with heart failure by reducing intravascular fluid load and minimizing the risk of acute decompensation.

We observed low rates of AF recurrence at 12 months in both groups (16.3% in the vHPSD group and 13.9% in the standard group), consistent with previously reported recurrence rates ranging from 10 to 17% (7, 10, 11, 17). While one study using 70-watt RF applications with the FlexAbility catheter, reported fewer arrhythmia recurrences at one year with higher-energy ablation (6), most studies evaluating 90-watt, 4-second RF applications with the QDOT Micro catheter have demonstrated comparable clinical efficacy between vHPSD and standard-power techniques (5), aligning with our findings. The use of different sheath types (deflectable vs. fixed) between groups may represent an important limitation when comparing the two ablation strategies, as it is difficult to fully separate the independent contribution of the ablation system and the sheath to procedural and clinical outcomes. Although some evidence suggests that deflectable sheaths may improve catheter stability and potentially influence procedural outcomes, including AF recurrence, this should be interpreted with caution, as most available data derive from observational studies, while the limited randomized controlled trial are relatively small and have yielded inconsistent results (18). Importantly, acute procedural success was comparable between groups in our cohort, suggesting that sheath type alone is unlikely to explain the observed clinical outcomes. Ideally, a randomized trial comparing vHPSD and standard-power ablation with uniform use of a deflectable sheath in both arms would provide a more controlled assessment of the independent contribution of ablation energy to long-term outcomes. Our findings should therefore be interpreted as a pragmatic, real-world comparison, reflecting current clinical practice.

Regarding longer-term outcomes, Kaplan–Meier analysis did not show any signal of a difference in AF recurrence between groups throughout the available follow-up period. Nevertheless, given that the median follow-up was significantly different between groups (vHPSD: 554 days; standard: 728 days; *p* < 0.0001) and was shorter than 24 months in the vHPSD group, the 2-year estimates should be interpreted with caution.

Despite the non-significant trend toward fewer ED visits due to AF or CHF in the vHPSD group, the healthcare utilization was comparable between groups at 12 months. None of the patients required hospitalization for CHF, AF, ACS, or stroke, possibly reflecting both the effectiveness of the procedure and the overall good health status of the population included in this study. In fact, this was a relatively young cohort (median age 62.8 years, IQR 54.2, 68.8), with a median haemoglobin level of 14.7 g/dL (IQR 13.5, 15.6), preserved renal function (median serum creatine level 0.8 mg/dL, IQR 0.8, 1.0), and a median ejection fraction of 59% (IQR 56, 63), suggesting a low comorbidity burden. There were also no significant differences between groups regarding the need for new antiarrhythmic interventions at 12 months, with similarly low rates of AADs reintroduction, electrical cardioversion, and redo procedures.

Although QoL can be a subjective outcome, assessing symptoms and their impact on patients’ daily life remains a key objective, especially for younger or more physically active patients, representing an important therapeutic target in the management of AF (1, 3). In our cohort, patients in the standard power group tended to have higher AFEQT scores at both baseline and 12 months. Nevertheless, both groups exhibited significant and similar improvements in QoL measurements, following the procedure. As most documented AF recurrences were symptomatic, lowering AF burden is likely to result in better symptom control and improved QoL. Our findings, consistent with previously published studies (19–21), demonstrated that a rhythm control strategy using catheter ablation led to clinically significant improvements in patients’ QoL, regardless of RF type.

The use of digital platforms for patient follow-up may facilitate earlier detection and management of arrhythmia recurrences (22). In our study, the high response rates to the quality-of-life questionnaires support the feasibility of incorporating digital health tools into routine AF care.

Previous studies have established the safety of AF catheter ablation (4, 5). In our study, both procedures demonstrated a favourable safety profile, with a low prevalence of adverse events. In the standard group, adverse events included one vascular complication (femoral pseudoaneurysm) and one case of pericarditis. In the vHPSD group, one case of pericardial effusion was documented. None of the reported events were life-threatening or required invasive intervention. A recent systematic review documented a very low prevalence of vascular complications (1.3%) and pericardial effusion/tamponade (0.8%) (23), aligning with our findings. This study was underpowered to detect rare complications such as esophageal lesions or atrioesophageal fistulas, which have an estimated incidence of less than 0.1% (2, 24). However, by producing wider and more superficial lesions, vHPSD ablation may offer a protective effect against such injuries. Two previous trials (13, 25) have suggested a trend to higher rates of cerebral embolism with vHPSD compared to standard-power ablation. In our cohort, asymptomatic cerebral lesions were not routinely screened during follow-up; however, no symptomatic neurologic events were observed in either group.

### 4.1 Limitations

This study was conducted in a high-volume, experienced centre, with a structured two-year follow-up program, providing valuable insights into the safety and long-term effectiveness of vHPSD AF ablation. However, some limitations should be acknowledged. First, the retrospective, non-randomized design inherently limits causal inference. Second, the inclusion of cohorts from different time periods may introduce time-period bias. Nevertheless, both PVI strategies are technically comparable, all procedures were performed by the same experienced operators and therefore, the learning curve associated with the implementation of vHPSD was considered minimal. No major changes in institutional protocols or peri-procedural management occurred during the study period and patient selection was not intentionally different between periods. Propensity score matching was performed, to mitigate potential confounding, resulting in well-balanced baseline characteristics between groups. Despite these considerations, unmeasured temporal confounders cannot be entirely excluded. Third, the use of different sheath types between groups may represent a potential confounding factor when comparing the two ablation strategies, as previously discussed. Fourth, AF recurrence was assessed through intermittent monitoring, which may have underestimated the true arrhythmia burden, although this limitation is unlikely to have differentially affected the two groups. Finally, the single-center nature of the study and the relatively young and healthy cohort may limit the generalizability of the findings.

## 5 Conclusion

VHPSD AF ablation proved to be a more efficient technique with a shorter procedural time, achieving similar clinical outcomes despite having a lower FPI rate. Both procedures appeared to be safe with low prevalence of adverse effects.

## Data Availability

The raw data supporting the conclusions of this article will be made available by the authors, without undue reservation.
