# Supplementary material for: Clinical and procedural outcomes of very high-power short-duration vs. standard power ablation for pulmonary vein isolation
Source: Front Cardiovasc Med. 2026 Apr 21;13:1806509. doi: 10.3389/fcvm.2026.1806509 (PMC13139015; doi:10.3389/fcvm.2026.1806509)
Supplement: Supplementary file 1 [file Datasheet1.docx]

Supplementary Material

# Study flowchart

**
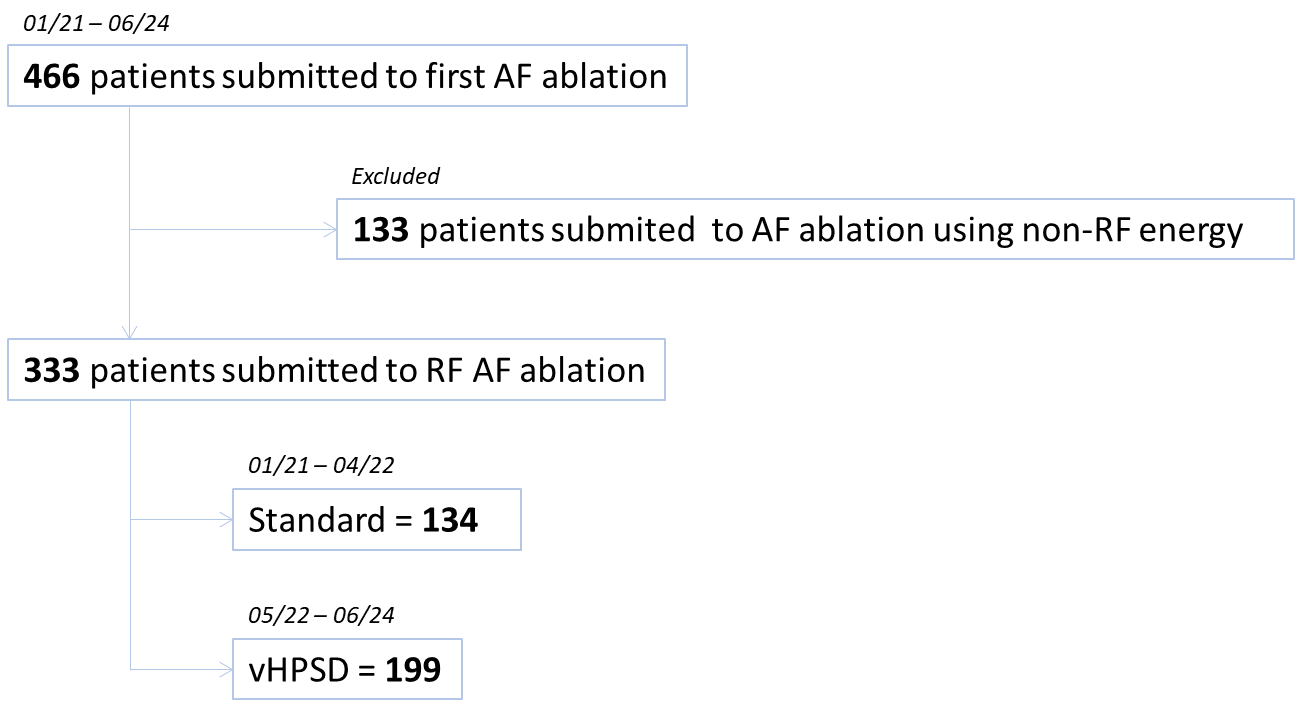
**

**Supplementary Figure 1.** Study flowchart. AF: atrial fibrillation, RF: radiofrequency; vHPSD: very high-power short-duration

# Propensity-score matching analysis

To address the problem of performing this analysis with missing data (**Supplementary Figure 2**), we assumed the missing values were missing at random. We performed multiple imputations by chained equations, with predictive mean matching for numerical data and logistic regression for categorical data (mice R package). This method preserves the inherent structure of the data and reduces the bias that could arise from simple imputation methods.

The a priori variable selection for propensity score matching included known predictors of clinical outcomes after atrial fibrillation ablation from prior studies. It included 19 variables: age, gender, arterial hypertension, body mass index, smoking history, sleep apnea, diabetes mellitus, cerebrovascular disease, coronary heart disease, chronic kidney disease, systolic dysfunction, moderate or severe valvular heart disease, dilated left atrium, implanted cardiac device, oral anticoagulant type, antiarrhythmic therapy, number of previous electrical cardioversions, atrial fibrillation type and time since diagnosis. To search for residual confounding, we performed univariate analysis for all variables associations with the radiofrequency group allocation, adding the following three variables: dyslipidaemia, beta-blocker therapy and intraprocedural cardioversion. A propensity score-based 1:1 match was performed with the nearest-neighbor method (caliper width of 0.2 SD of the logit). The absolute between-group standardized mean difference was veriﬁed for all baseline variables, and it was below 0.1 (**Supplementary Figure 3**). We ended up with 226 matched patients, 86 unmatched in the vHPSD group, and 21 in the standard-power group (**Supplementary Figure 4**).


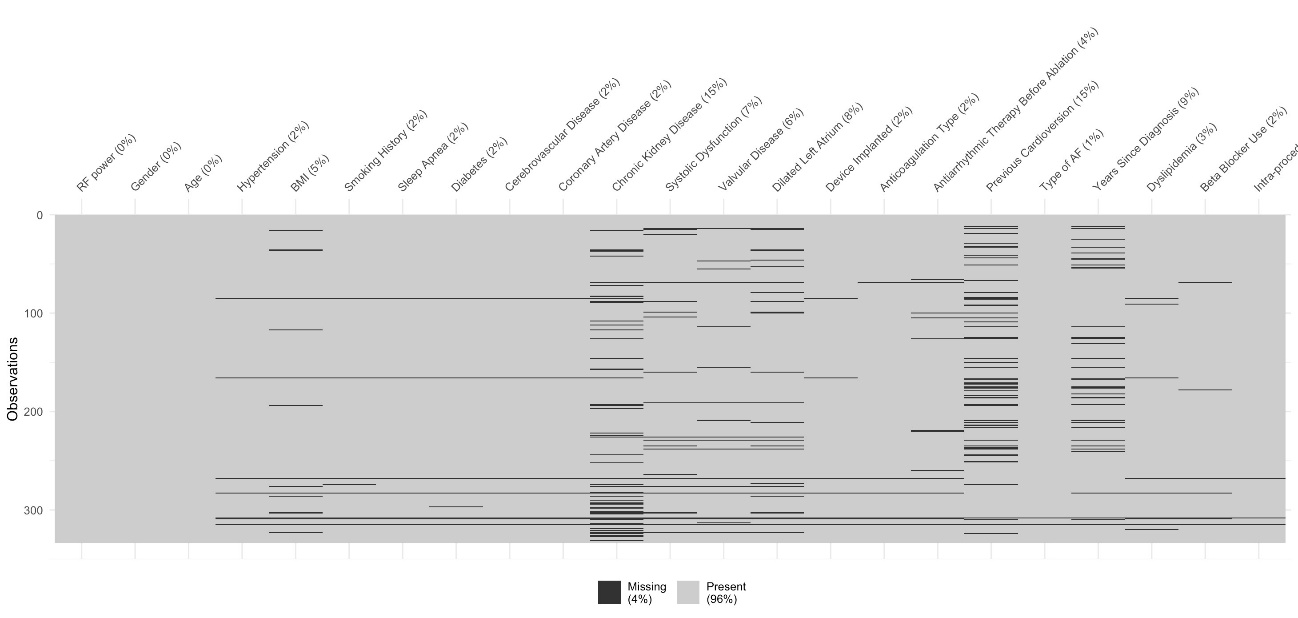


**Supplementary Figure 2.** Missing map of features selected for the propensity score matching analysis.


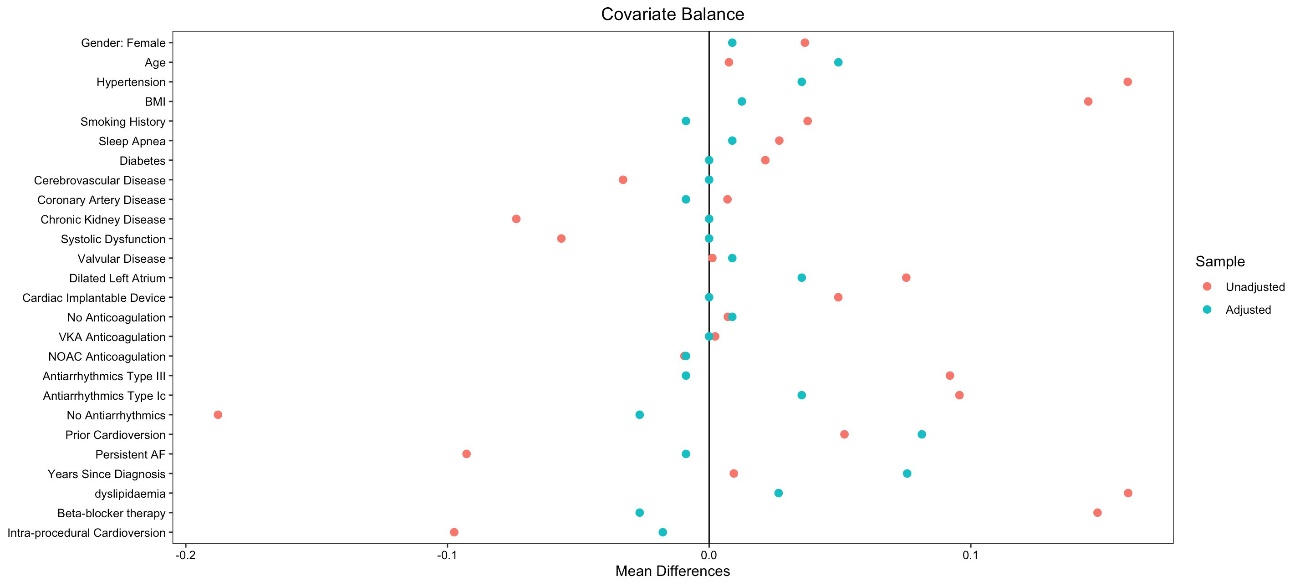


**Supplementary Figure 3.** Standardized mean differences for the propensity score variables before matching (Unadjusted) and after the matching (Adjusted). The love plot shows <0.1 standardized mean differences in all baseline variables.


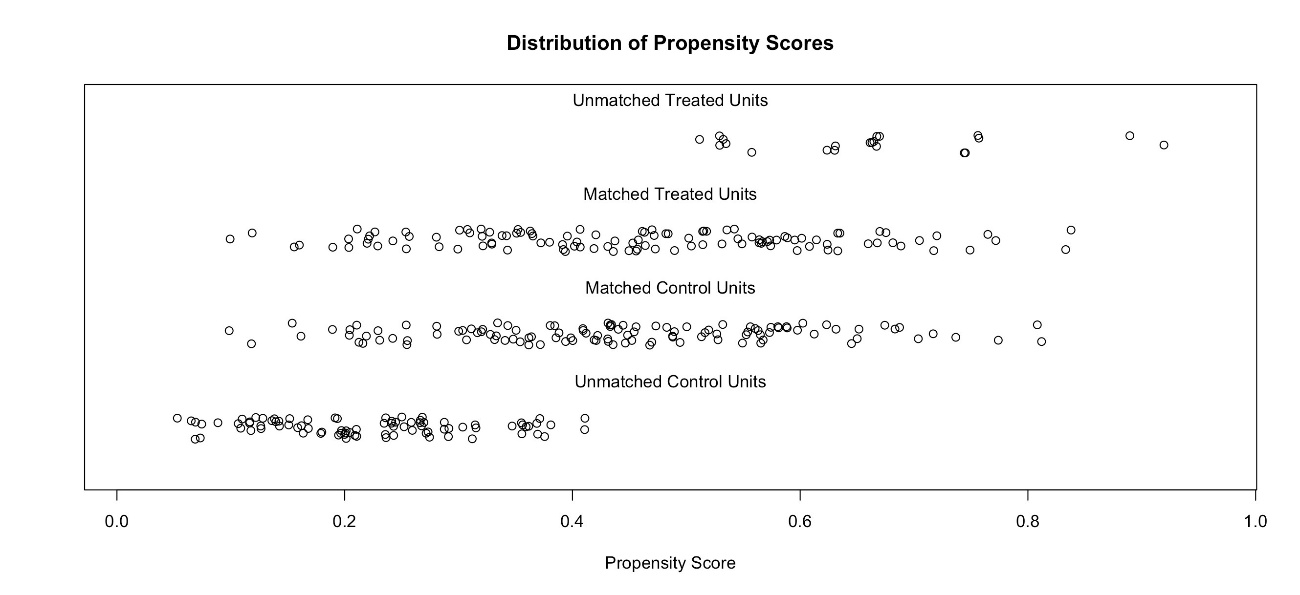


**Supplementary Figure 4.** Jitter plot showing similar distributions of propensity scores for treated and control groups.

## Sensitivity analysis: propensity score matching including calendar time

As a sensitivity analysis to further address potential chronological confounding, we re-estimated the propensity scores including calendar time as an additional covariate. Calendar time was defined as the number of days since the first AF ablation procedure in the study period, derived from the index procedure date. The propensity score model included the same baseline clinical variables as in the main with the addition of calendar time. 1:1 nearest-neighbour matching with a caliper of 0.2 on the logit of the propensity score was then repeated. Due to the complete temporal separation between standard-power and vHPSD ablation, overlap in propensity scores was markedly reduced and only 84 patients (42 per group) could be matched. Covariate balance in this restricted cohort remained acceptable, with standardized mean differences generally below 0.2 for baseline variables and a modest difference in calendar time (mean 545 vs. 592 days).

## Sensitivity analysis: clinical efficacy outcomes

In this time-adjusted matched sample, there was still no statistically significant difference between groups in the main time-to-event outcomes. At 12 months, estimated freedom from AF/atrial tachyarrhythmia recurrence was 77.7% in the vHPSD group and 86.9% in the standard-power group (marginal HR 0.70, cluster-robust standard error of 0.41, p=0.390). Freedom from healthcare utilization at 12 months was 90.2% in the vHPSD group and 90.1% in the standard-power group, with a marginal hazard ratio of 0.63 (cluster-robust standard error of 0.51, p = 0.36). For new antiarrhythmic interventions, freedom from events at 12 months was 92.3% in the vHPSD group and 89.5% in the standard-power group, with a marginal hazard ratio of 0.69 (cluster-robust standard error of 0.60, p = 0.53), again showing no statistically significant difference.

# Quality-of-Life data

**Supplementary Table 1.** Estimated marginal means and mean adjusted differences of AFEQT scores between baseline and 12 months in both groups.

| Quality-of-Life Questionnaires | Baseline | 12 months | Mean Adjusted Difference (95% CI) | *p*-value |
| --- | --- | --- | --- | --- |
| Mean overall AFEQT score (se) | 67.4 (1.4) | 81.5 (1.7) | +13.9 (10.4 – 17.5) | <0.001 |
| vHPSD | 65.1 (1.9) | 77.0 (2.6)* | +11.9 (6.6 – 17.2) | <0.001 |
| Standard | 70.3 (3.1) | 85.4 (2.3)* | +15.1 (10.3 – 19.8) | <0.001 |

*Statistically significant between-group differences at each time point. AFEQT score: Atrial Fibrillation Effect on QualiTy-of-life score; CI: confidence interval; SE: standard error; vHPSD: very high-power short-duration

# Safety data

**Supplementary Table 2.** Safety profile and adverse effects.

| Variable | vHPSD  (n=113) | Standard  (n=113) | *p*-value |
| --- | --- | --- | --- |
| Vascular complications | 0 (0) | 1 (1.0) | 1 |
| Other complication   - Pericarditis - Pericardial effusion - Phrenic nerve palsy - Stroke/Transient ischemic attack - Atrioesophageal fistula | 0 (0)  1 (0.9)  0 (0)  0 (0)  0 (0) | 1 (0.9)  0 (0)  0 (0)  0 (0)  0 (0) | 0.872 |

All results are presented as n (%). vHPSD: very high-power short-duration.
